# Soil microbial community composition and nitrogen enrichment responses to the operation of electric power substation

**DOI:** 10.3389/fmicb.2024.1453162

**Published:** 2024-08-20

**Authors:** Zhi-Xin Xu, Bo Zeng, Sheng Chen, Sa Xiao, Lin-Gao Jiang, Xiang Li, Yun-Fang Wu, Le-Xing You

**Affiliations:** ^1^High Voltage Branch of State Grid Fujian Electric Power Co., Ltd., Fuzhou, China; ^2^College of Geography and Environmental Sciences, Zhejiang Normal University, Jinhua, China

**Keywords:** soil microbial community, electric power substation, nitrogen processing, distance, metagenomic sequencing technique

## Abstract

The surge in global energy demand mandates a significant expansion of electric power substations. Nevertheless, the ecological consequences of electric power substation operation, particularly concerning the electromagnetic field, on soil microbial communities and nitrogen enrichment remain unexplored. In this study, we collected soil samples from six distinct sites at varying distances from an electric power substation in Xintang village, southeastern China, and investigated the impacts of electromagnetic field on the microbial diversity and community structures employing metagenomic sequencing technique. Our results showed discernible dissimilarities in the fungal community across the six distinct sites, each characterized by unique magnetic and electric intensities, whereas comparable variations were not evident within bacterial communities. Correlation analysis revealed a diminished nitrogen fixation capacity at the site nearest to the substation, characterized by low moisture content, elevated pH, and robust magnetic induction intensity and electric field intensity. Conversely, heightened nitrification processes were observed at this location compared to others. These findings were substantiated by the relative abundance of key genes associated with ammonium nitrogen and nitrate nitrogen production. This study provides insights into the relationships between soil microbial communities and the enduring operation of electric power substations, thereby contributing fundamental information essential for the rigorous environmental impact assessments of these facilities.

## 1 Introduction

The relentless advance of modernization has brought about an increasing demand for electric energy. This demand necessitates the efficient transportation of electricity from power generation facilities. This intricate process relies on a vast network comprising power substations, transmission lines, and distribution lines. Among them, power substations serve as crucial installations of equipment designed for voltage control and adjustment (Biasotto and Kindel, 2018). The Chinese landscape reflects a proliferation of power substations and the extensive deployment of electrical power lines, traversing diverse terrains such as farmland and highways. This remarkable infrastructural landscape is, however, compelled to evolve in response to the escalating need for electricity, necessitating the establishment of additional electric power substations. Nevertheless, it is essential to recognize that electric power substations, throughout their construction and operational phases, pose potential environmental considerations (Bagli et al., 2011).

In many countries, rigorous vegetation management is required in the vicinity beneath power cables to avert interference with line structural integrity and energy transmission. Furthermore, the operation of power lines and electrical equipment engenders the generation of electromagnetic fields (Biasotto and Kindel, 2018). As crucial components of terrestrial ecosystems, soil inevitably bear the imprint of electric power substations. Soil microorganisms, as guardians of soil health, regulate soil nutrient cycles, including the vital nitrogen cycle, and exert a profound influence on plant productivity (Gadd, 2010; Faust and Raes, 2012; Whalen et al., 2022). In response to environmental stressors, soil microorganisms exhibit distinctive adaptive responses, such as the production of diverse enzymes like superoxide dismutase (SOD), which provide effective resistance against the harmful effects of reactive oxygen species (Roubalová et al., 2018) and safeguards plant well-being (Huseynova et al., 2014). Despite substantial progress in environmental impact assessment pertaining to electric power substations, notable gaps persist in representing distinct biological strata, such as genes, and integrating a wide range of biodiversity metrics encompassing composition, structure, and function into impact prediction (Biasotto and Kindel, 2018). Of particular note is the dearth of scholarly exploration into the effects of electromagnetic fields generated by electric power substations on soil microorganisms, despite the uncertain biological implications at times (Beretta et al., 2019).

Aligned with the aforementioned context, the principal objective of this study was to delineate the effects of magnetic induction intensity (MII) and electric field intensity (EFI) emanating from the operation of electric power substations on soil physicochemical properties, alongside the structural and functional aspects of microbial communities across and shifts in functional genes related to nitrogen cycling. A specific focus was placed on elucidating correlations between soil physicochemical properties and microbial communities, as well as genes associated with the nitrogen cycle in the proximity of the substation.

## 2 Materials and methods

### 2.1 Site description and soil sample collection

**Figure 1:**
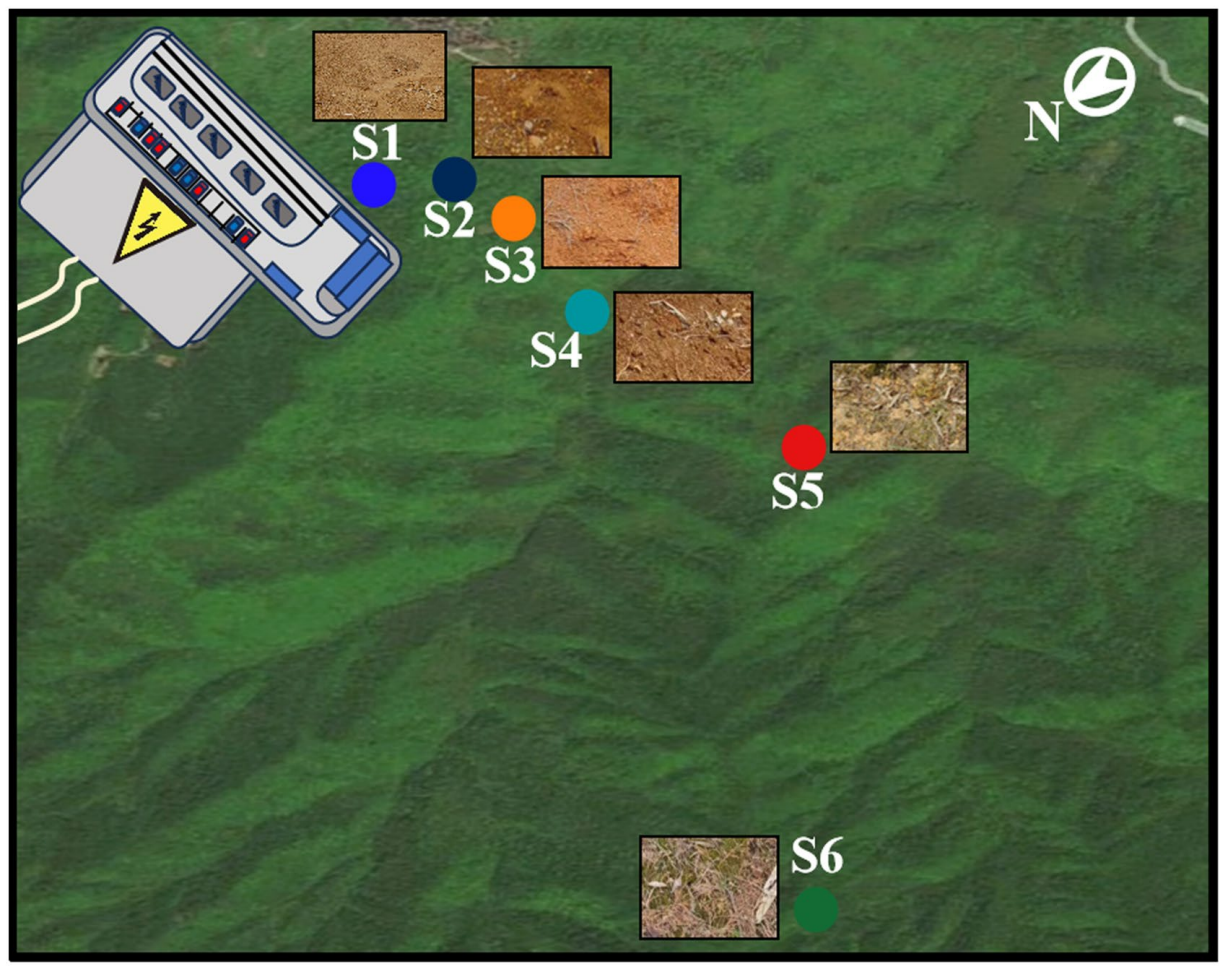
Schematic of soil collection sites distance from an electric power substation.

Soil specimens were systematically obtained from the environs of an electric power substation (1,000 kV, 16.3 hm^2^) located at coordinates 119°08′N latitude and 26°34′E longitude, situated within Xintang village, Minhou county, Fujian province, southeastern China (as delineated in Supplementary Table S1). Xintang village occupies a locale characterized by a subtropical oceanic monsoon climate, typified by annual mean temperatures ranging between 17 and 21°C, and annual average precipitation levels spanning 1,400 to 2000 mm. The topography of the study site comprises a modest, low-lying basin with predominant rainfall occurring between May and September. Soil samples were diligently collected from distinct designated regions within the 0–20 cm stratum of the topsoil (Figure 1). It is noteworthy that the selected areas exhibited minimal vegetation cover. For each of the sampling locales (denoted as S1 through S6), an array of triplicate sampling plots, each measuring 2 meters by 2 meters, was judiciously established in a randomized fashion. Within the confines of each sampling plot, three discrete soil samples were haphazardly extracted (Guo et al., 2020; Chen et al., 2023), sealed within aseptic ziplock bags and expeditiously transported, under cryogenic conditions facilitated by dry ice, to the laboratory facility. Each soil sample underwent a meticulous sieving process, utilizing a 2 mm mesh screen, with the dual objectives of homogenizing the soil matrix and eliminating larger particles, soil-dwelling organisms, and vegetative debris.

### 2.2 Soil physicochemical properties measurement

Fresh soil (100 g) was collected in each designated sampling plot using ring knives to determine soil bulk density, a parameter assessed through weight method (Zhang et al., 2023). The determination of soil dry matter and moisture content was undertaken employing the classical gravimetric method, while soil particle density was ascertained through the volume replacement technique. The quantification of soil organic matter (SOM) content was executed following the classical potassium dichromate method. The MII and EFI of sampling sites were measured using a Low-frequency electromagnetic field analyzer (Narda EFA-300, Germany). Moreover, the pH value of each soil sample, prepared at a soil-water ratio: 1:2.5, was diligently measured utilizing a pH meter (Sartorius PB-10, Germany). Specifically, for the determination of nitrite nitrogen (NO_2_^−^-N) and ammonium nitrogen (NH_4_^+^-N) concentrations, the soil samples (soil-water ratio: 1:5) were intimately mixed with KCl solution at a final concentration of 1 M. Subsequently, this mixture underwent agitation at 200 rpm for a duration of 1 h and was then subjected to centrifugation (3,000 rpm, 10 min) to facilitate the retrieval of the supernatant. The contents of NO_2_^−^-N and NH_4_^+^-N within the supernatant were subsequently quantified utilizing a UV–vis spectrophotometer (TU-1810, Bejing Purkinje General Instrument Co. Ltd., China) (see details in Supplementary Section S1), in strict accordance with their respective standard curves. The content of nitrate nitrogen (NO_3_^−^-N) within the filtered supernatant which was determined using a flow injection auto-analyzer (Skalar Analytical, AACE, Germany) (Chen et al., 2023). Furthermore, the overall content of total nitrogen (TN), soil organic nitrogen (SON), total carton (TC) and total organic carbon (TOC) was comprehensively determined (see Supplementary Sections S1, S2).

### 2.3 Determination of enzyme content in soil

The levels of SOD, malondialdehyde (MDA), glutathione (GSH), lactate dehydrogenase (LDH), acid protease, acid phosphatase and soil sucrase were quantified utilizing an enzyme assay kit (Gene Hunter, HongKong, China) (see the details in Supplementary Section S3) to evaluate the response of soil microorganisms to environmental stressors, including electromagnetic fields.

### 2.4 Soil DNA extraction and sequencing

Microbial genomic DNA were extracted from individual soil samples (0.5 g) using the Omega E.Z.N.A Stool DNA Kit for Soil (Omega Bio-tek, Inc., United States), following the manufacturer’s instructions. The purity and quality of the genomic DNA were assessed via 1% agarose gels electrophoresis, and their concentration were accurately quantified using the Qubit 4.0 Fluorometer (Thermo Fisher Scientific Inc., United States). Subsequently, the DNA was fragmented to 300 bp employing the Covaris ultrasonic crusher, and the resulting fragments underwent further processing, including end repair, A tailing, and ligation of Illumina compatible adapters. Finally, sequencing were conducted on an Illumina NovaSeq PE150 platform at Allwegene Company (Beijing, China) (Supplementary Table S2; Supplementary Section S4). The Illumina NovaSeq sequencing data were deposited in the Sequence Read Archive (SRA) database of the National Center for Biotechnology Information (NCBI), under accession number PRJNA1037611 (SUB13936859). The abundance of functional genes relevant to nitrogen metabolism were examined based on sequencing data combined with Kyoto Encyclopedia of Genes and Genomes (KEGG) database.

### 2.5 Statistical analyses

Significant differences were assessed through one-way analysis of variance (SPSS 19) with *ρ <* 0.05 considered as statistically significant. Non-metric multidimensional scaling (NMDS) and principal coordinate analysis (PCoA) based on the Bray–Curtis dissimilarity were conducted using the R (Version 4.0.2) package ggplot2. Vegan ggplot2 package was employed for redundancy analyses (RDA) to analyze the correlation between environmental factors and the abundance of microbial community phyla. A Pearson correlation test was executed to examine the relationships between soil physicochemical properties and the relative abundance of microbial community, utilizing the R (Version 3.6) packages “psych” and “pheatmap.”

## 3 Results

### 3.1 Soil physicochemical properties of six different collection sites

The physicochemical characteristics of soil samples from each collection site were comprehensively detailed in Table 1. Variations in soil pH were observed across locations, ranging from 4.9 to 7.1, while SOM and bulk density spanned from 8.84 to 27.25 g·Kg^−1^ and 1.01 to 1.51 g·cm^−3^, respectively. Both MII and EFI exhibited the highest values at the S1 site, diminishing with increasing distance from the electric power substation The TC and TOC content in the soil samples from S1 were lower than those at other collection sites, with the TN content (5.50 g·Kg^−1^) at S1 surpassing that observed at other sites. The NO_3_^−^-N content at the S1 and S2 sites exceeded that at the remaining sampling sites. Conversely, for NH_4_^+^-N, the lowest content was observed at the S1 site compared to other collection points, with its content being lower than that of NO_3_^−^-N. Furthermore, we assessed several types of soil enzyme content as key indicators for microbial functioning controlling the decomposition rate of SOM and nutrient cycling processes, evaluating the influence of the operation of the electric power substation on the soil. The levels of MDA, SOD, GSH, LDH, acid protease, acid phosphatase and soil sucrase in the soil samples from S1 were marginally higher than those at other collection sites (Table 1).

**Table 1:** Soil physicochemical properties and soil enzymes content from each collection site.

Site	S1	S2	S3	S4	S5	S6
pH	7.1	4.9	5.1	5.1	5.0	5.9
SOM (g·Kg^−1^)	10.69 ± 0.04	13.77 ± 0.33	13.46 ± 0.33	27.25 ± 0.54	8.84 ± 0.12	12.65 ± 0.28
Soil bulk density (g·cm^−3^)	1.51 ± 0.020	1.26 ± 0.006	1.19 ± 0.009	1.01 ± 0.003	1.13 ± 0.027	1.30 ± 0.014
Soil density (g·cm^−3^)	1.66 ± 0.019	1.50 ± 0.029	2.61 ± 0.007	1.76 ± 0.033	1.25 ± 0.009	1.57 ± 0.048
Soil moisture content (%)	26.91%	31.58%	31.18%	32.09%	32.82%	28.65%
Magnetic induction intensity (μT)	0.43 ± 0.024	0.18 ± 0.017	0.15 ± 0.014	0.073 ± 0.009	0.034 ± 0.005	0.012 ± 0.020
Electric field intensity (kV·m^−1^)	1.47 ± 0.03	0.86 ± 0.03	0.69 ± 0.02	0.24 ± 0.01	0.001 ± 0.00	0.000
TC (g·Kg^−1^)	7.51 ± 0.25	9.85 ± 0.03	8.45 ± 0.41	15.49 ± 1.01	7.85 ± 0.80	9.12 ± 0.79
Solid TOC (g·Kg^−1^)	5.60 ± 0.23	7.52 ± 0.31	6.61 ± 0.07	12.31 ± 0.21	6.43 ± 0.53	6.55 ± 0.19
NO_2_^−^ (μg·Kg^−1^)	2.86 ± 0.04	1.56 ± 0.04	0.68 ± 0.04	0.75 ± 0.04	2.55 ± 0.04	2.11 ± 0.00
NH_4_^+^-N (mg·Kg^−1^)	3.49 ± 0.10	16.22 ± 2.79	28.16 ± 0.31	40.21 ± 0.46	3.84 ± 0.27	15.17 ± 0.10
NO_3_^−^-N (mg·Kg^−1^)	8.87 ± 0.12	8.77 ± 0.13	6.69 ± 0.06	6.48 ± 0.12	3.88 ± 0.07	8.64 ± 0.22
SON (mg·Kg^−1^)	5486.26 ± 132.51	5162.99 ± 121.46	1153.11 ± 61.31	4281.37 ± 78.34	785.93 ± 25.41	2985.51 ± 45.54
TN (mg·Kg^−1^)	5498.63 ± 122.41	5188.19 ± 117.95	1187.97 ± 60.40	4328.08 ± 81.24	793.66 ± 26.21	3009.32 ± 51.94
MDA (nmol·L^−1^)	9.46 ± 0.16	9.17 ± 0.33	9.40 ± 0.31	8.41 ± 0.32	8.19 ± 0.52	8.05 ± 0.07
SOD (U·mL^−1^)	21.08 ± 0.97	20.47 ± 0.21	16.54 ± 0.12	17.67 ± 0.38	18.96 ± 0.47	16.90 ± 1.04
GSH (ng·L^−1^)	58.14 ± 1.51	58.10 ± 2.26	55.70 ± 2.73	51.08 ± 2.02	45.40 ± 1.16	49.79 ± 2.21
LDH (IU·L^−1^)	62.09 ± 1.50	56.78 ± 2.00	46.81 ± 2.67	44.25 ± 3.71	47.70 ± 0.86	53.15 ± 1.85
Acid protease (U·L^−1^)	267.04 ± 12.80	227.70 ± 9.93	234.43 ± 9.18	218.77 ± 15.88	209.46 ± 16.75	224.85 ± 3.00
Acid phosphatase (IU·L^−1^)	53.04 ± 2.66	40.94 ± 2.30	51.43 ± 2.78	47.26 ± 1.19	46.36 ± 1.27	48.00 ± 2.25
Soil Sucrase (U·L^−1^)	1066.92 ± 45.93	994.84 ± 43.49	853.59 ± 58.70	942.78 ± 53.68	857.25 ± 50.14	904.36 ± 72.36

### 3.2 Soil microbial characteristics of six different collection sites

**Figure 2:**
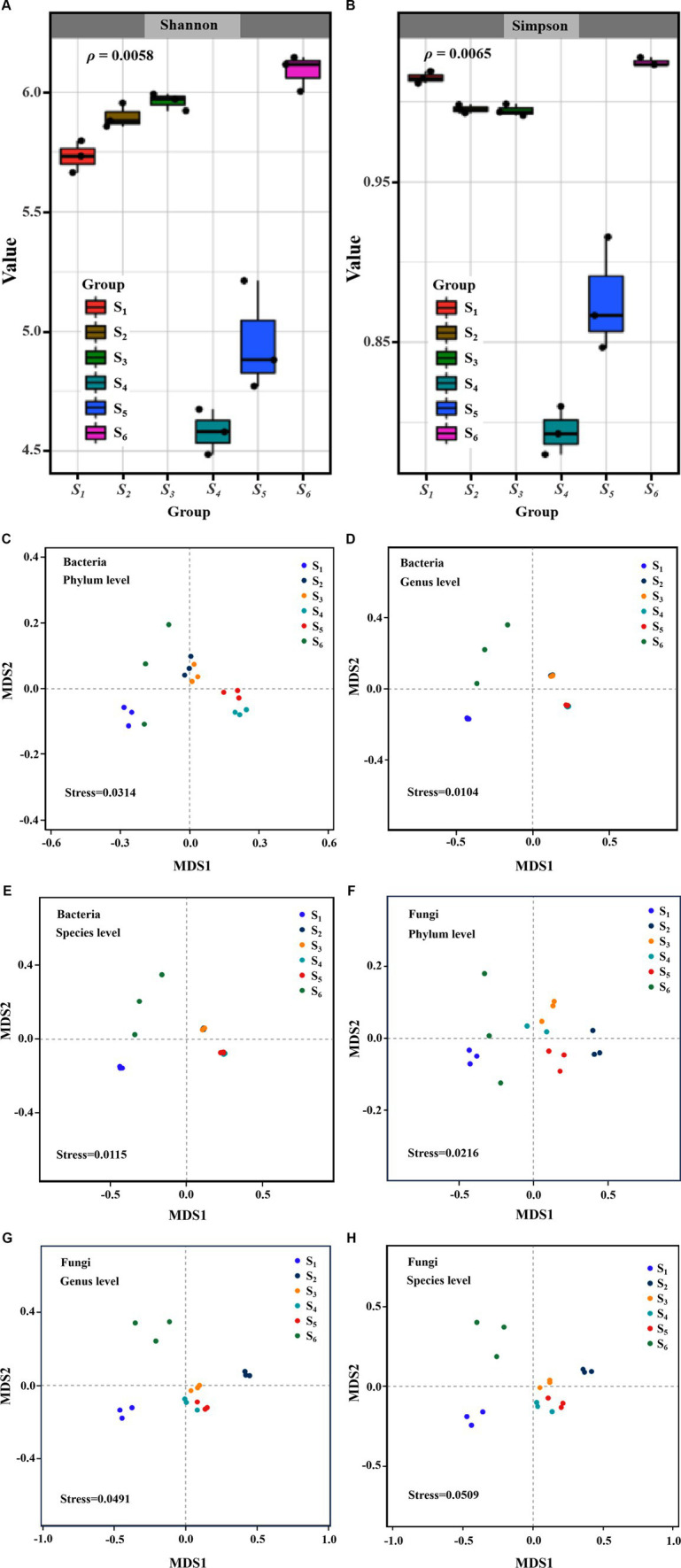
Alpha diversity Shannon **(A)** and **(B)** Simpson index of soil microbial communities collected from 6 points distance from an electric power substation; NMDS analysis of the differences in bacterial and fungus communities structures at phylum **(C,F)**, genus **(D,G)** and species **(E,H)** based on Bray-Curtis distance.

Metagenomic sequencing was conducted on these six sampling sites, yielding an average of 7.21 million reads per sample (Supplementary Table S2). From these metagenomes, 5,694,546 non-redundant catalog genes were identified, characterized by an average length of 637.2 bp. Among them, there were 1,648,487 genes annotated by KEEG with an annotation rate of 0.289, suggesting that the existence of numerous genes with functions yet to be elucidated. Representative sequences of the non-redundant gene catalog were annotated using the NCBI NR database (Version: 2021.11) through BLASTP implemented in Diamond (Buchfink et al., 2014) (http://www.diamondsearch.org/index.php, version 0.8.35), with an e-value cutoff of 1e^−5^ for taxonomic annotations. In the Initial phase of analysis, an alpha diversity assessment was undertaken to evaluate species richness within the soil samples. As illustrated in Figures 2A,B, a substantial difference in the richness and alpha diversity of soil microbes was evident among the 6 sites, as indicated by the Shannon index (*ρ* < 0.05) and Simpson index (*ρ* < 0.05). Notably, soil samples from S1 and S6, representing the sites nearest and furthest from the power substation, respectively, exhibited the highest values of Simpson index.

Furthermore, the analysis of beta diversity in soil microbial communities, employing NMDS and PCoA to elucidate differences in species composition, unveiled significant variation among the collection sites. Utilizing Bray–Curtis distance measurements, both NMDS (Figures 2C–E) and PCoA (Supplementary Figures S1A–C) highlighted noteworthy dissimilarities in the community composition of bacteria (stress<0.05; PERMANOVA, *ρ* = 0.001) across the 6 sites. The microbial communities of sites (S2–S3) and (S4–S5) exhibited tight clustering, indicating a relatively consistent microbial community composition at the S2 and S3 sites, as well as the S4 and S5 sites. However, sites S1 and S6 were distinctly separated, signifying inconsistency in the microbial community composition at these locations. In contrast, the fungal community structure did not demonstrate representativeness (stress>0.05) at the species level across the 6 sites (Figures 2F–H), but exhibited significant differences (PERMANOVA, *ρ* = 0.001) (Supplementary Figures S1D–F).

### 3.3 Soil microbial communities of six different collection sites

#### 3.3.1 Community composition of bacteria

**Figure 3:**
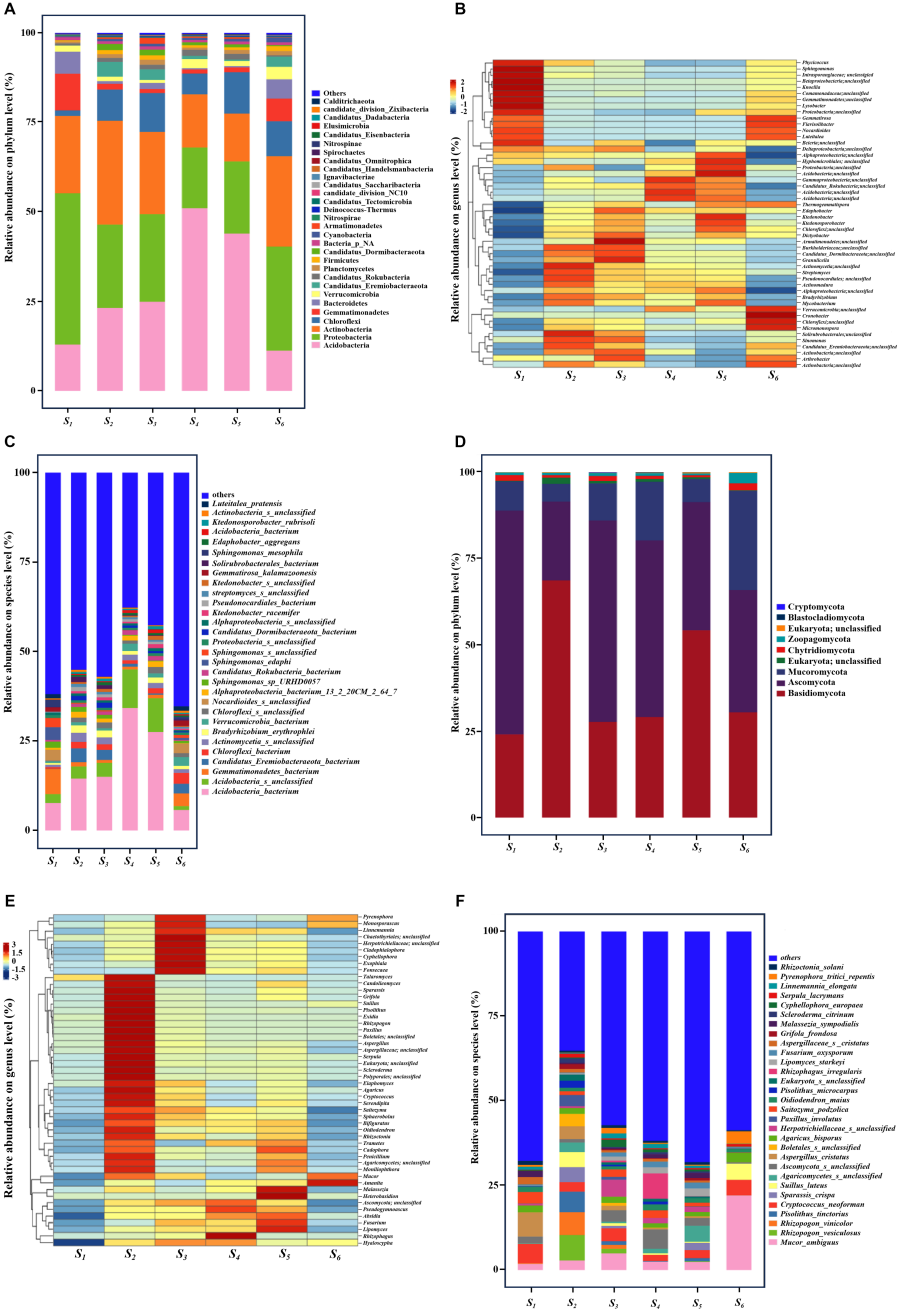
Composition of soil microbial communities in six collection sites. Composition of **(A–C)** bacteria and **(D–F)** fungal at the **(A,D)** phylum, **(B,E)** genus and **(C,F)** species level.

Bacterial phyla within soil samples from each collection site were analyzed at the phylum level (Figure 3A). Legends presenting relative abundance below the top 30 were excluded and categorized into other groups. The predominant bacterial populations, encompassing *Proteobacteria*, *Acidobacteria*, *Actinobacteria*, and *Chloroflexi*, collectively constituted over 65% of the total microbial population across all soil samples. The average relative abundance of Proteobacteria at the S1 site (42.64%) surpassed that of other sites. *Acidobacteria*, the most abundant bacterial phylum, exhibited reduced abundance at the S1 (13.03%) and S7 (11.31%) sites relative to other collection points. The phylum *Actinobacteria* attained its highest relative abundance at the S2 site (29.26%) and its lowest at the S5 site (13.48%). *Chloroflexi* exhibited notably lower relative abundance at the S1 site (1.55%) compared to the S5 (11.66%), S4 (5.89%), S3 (10.94%), S2 (8.77%) and S6 (9.86%) sites (Figure 3A). The relative notable enrichment of *Gemmatimonadetes* was observed at the S1 (10.35%) sites, exceeding that at the S5 (1.34%), S4 (1.15%), S3 (1.14%), S2 (1.60%) and S6 (6.34%).

The dissimilarities in soil microbial community composition across different sites were further elucidated through a detailed analysis employing a heatmap of bacteria genera, showcasing the top 50 species in overall abundances. As illustrated in Figure 3B, the relative abundances of these bacteria, including *Phycicoccus* (*Actinobacteria*), *Sphingomonas* (*Proteobacteria*), *Unclassified_Comamonadaceae* (*Proteobacteria*), *Knoellia* (*Actinobacteria*), *Unclassified_Gemmatimonadete*s, *Lysobacte*r (*Proteobacteria*), *Flavisolibacter* (*Bacteroidetes*), *Nocardioides* (*Actinobacteria*), *Luteitalea* (*Acidobacteria*), exhibited significantly higher abundances at the S1 site compared to other sites. At the species level (Figure 3C), *Sphingomonas mesophila* and *Sphingomonas edaphi*, individually constituting 2.72 and 4.85% of the total bacterial community, manifested a manifold increase in abundance at the S1 site relative to other sites. Notably, there was a heightened abundance of *Luteitalea_pratensis* at the S1 site.

#### 3.3.2 Community composition of fungi

Metagenomic sequence taxonomic analysis revealed nine phyla, with Basidiomycota, Ascomycota and Mucoromycota constituting the most abundant, collectively averaging over 95% of all sequences in the fungi community (Figure 3D). Ascomycota emerged as the most abundant phylum, with an average relative abundance exceeding 60% at the S1 sites and surpassing 50% at the (S3, S4) sites, while its relative abundance at the S2 site was comparatively lower. The relative abundance of *Basidiomycota* at the (S2 and S5) exceeded that at the other points, whereas *Mucoromycota* exhibited the highest relative abundance at the (S4 and S6) sites. The Heatmap of fungal genera with the top 50 species in overall abundances were presented in Figure 3E. The genera of detected fungal species, showing greater richness, displayed distinct distribution characteristics at the 6 sites. It was observed that a relative lower abundant was noted for most all species at the S1 site compared to other points. The most representative fungal genus was *Talaromyces* (*Ascomycota*) and *Aspergillus* (*Ascomycota*), individually constituting 4.63 and 12.75% of the total fungal community. Furthermore, Figure 3F delineated the distinct community composition at the species level for different collection sites. Notably, for the S6 site, the most representative species with greater abundance was *Aspergillus_cristatus*, which accounted for 8.16% of the total fungal community.

### 3.4 Nitrogen processing of soil microbial communities at different collection site

The functions annotated under KEGG level 1 for the 6 types of soil surrounding the electric power substation encompassed diverse categories: metabolism (ave. 51.73%), genetic information processing (ave. 14.59%), environmental information processing (ave. 13.08%), cellular processes (ave. 10.91%), human diseases (ave. 5.30%), and organic systems (ave. 4.39%) (Supplementary Figure S2A). Significantly enriched functional pathways at level 2 (>4%) included carbohydrate metabolism, amino acid metabolism, energy metabolism, metabolism of cofactors and vitamins, cellular community-prokaryotes, signal transduction, and membrane transport (Supplementary Figure S2B). Among these, the S1 site exhibited a higher proportion of amino acid metabolism, and a lower proportion of carbohydrate metabolism and cellular community-prokaryotes compared to all other sites. Relatively more abundant functional pathways at level 3 (>5%), such as two-component system, quorum sensing, ABC transporters, Oxidative phosphorylation, pyruvate metabolism, and ribosome, were observed across the 6 points (Supplementary Figure S2C), with no significant difference.

**Figure 4:**
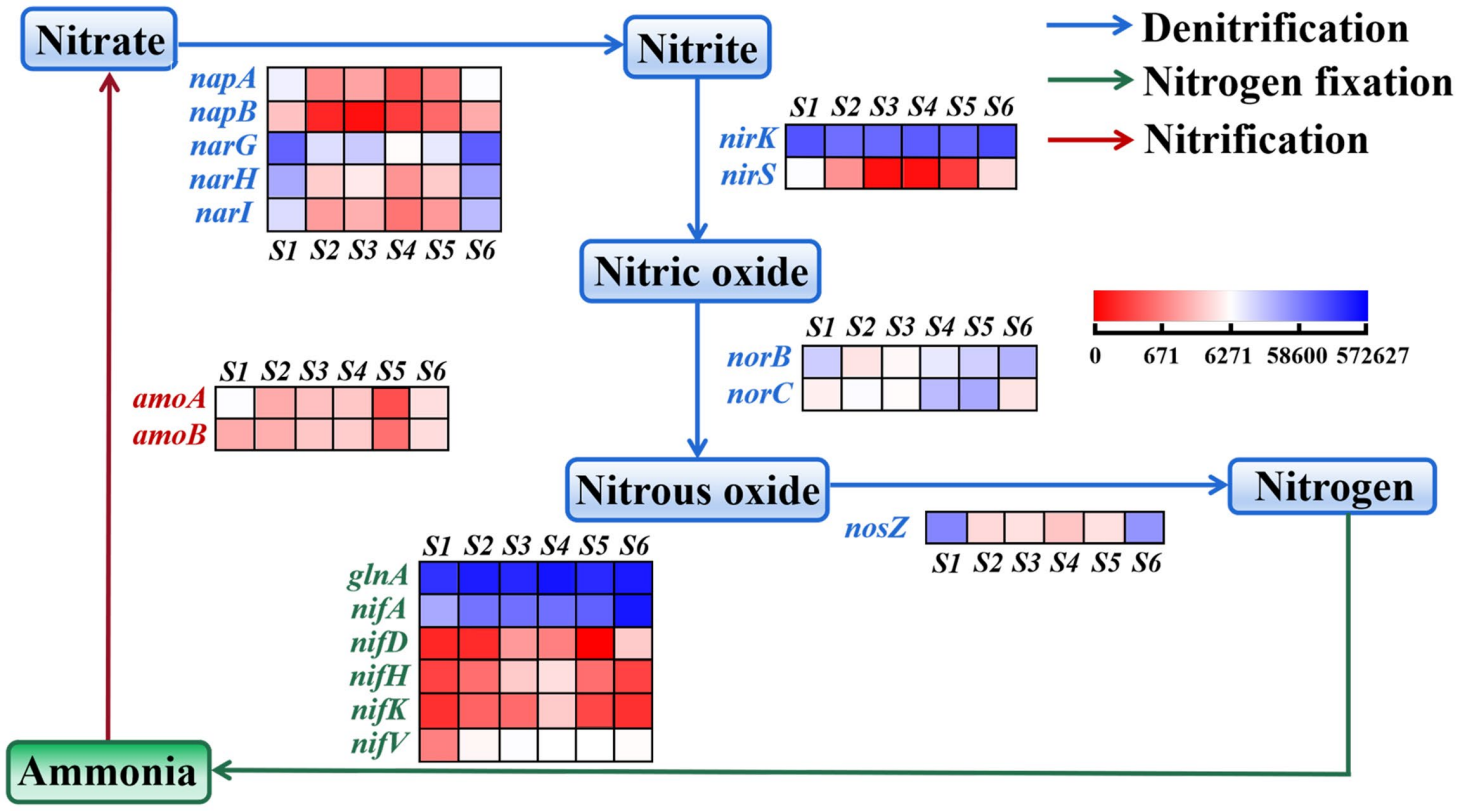
Heatmap representing the relative abundance of nitrogen functional genes in soil community in the S1–S6 samples.

Noteworthy were the nitrogen metabolism pathways, including nitrogen fixation, nitrification and denitrification. The relative abundance of the genes, such as *nifA, nifD*, *nifH*, *nifK*, and *nifV* (Kuypers et al., 2018; Bellés-Sancho et al., 2021), was lower at the S1 site than at other sites (Figure 4), suggesting a lower capacity for soil microbial nitrogen fixation (N_2_ → NH_3_). In contrast, the relative abundance of two genes, *amoA* and *amoB* (Wang et al., 2022), was higher at the S1 site than at other sites, indicating increased nitrification (NH_4_^+^ → NO_3_^−^). This observation aligns with the finding of low NH_4_^+^-N and high NO_3_^−^-N at the S1 site (Table 1). The total abundance of nitrogen fixation genes exhibited and increase with distance from the electric power substation (from S1 to S4), followed by a decline at S5 and S6 sites primarily attributed to the lower content of SOM, findings that corresponded with NH_4_^+^-N levels. Furthermore, the relative abundance of genes associated with NO_2_^−^, nitric oxide and nitrous oxide, as reported by Li et al. (2023), generally mirrored the levels of NO_2_^−^ in each soil sample, with the relative abundances of *nirK*, *nirS*, *norB*, *norC* and *nosZ* also depicted in Figure 4.

### 3.5 Correlation between soil microbial communities and environmental factors

**Figure 5:**
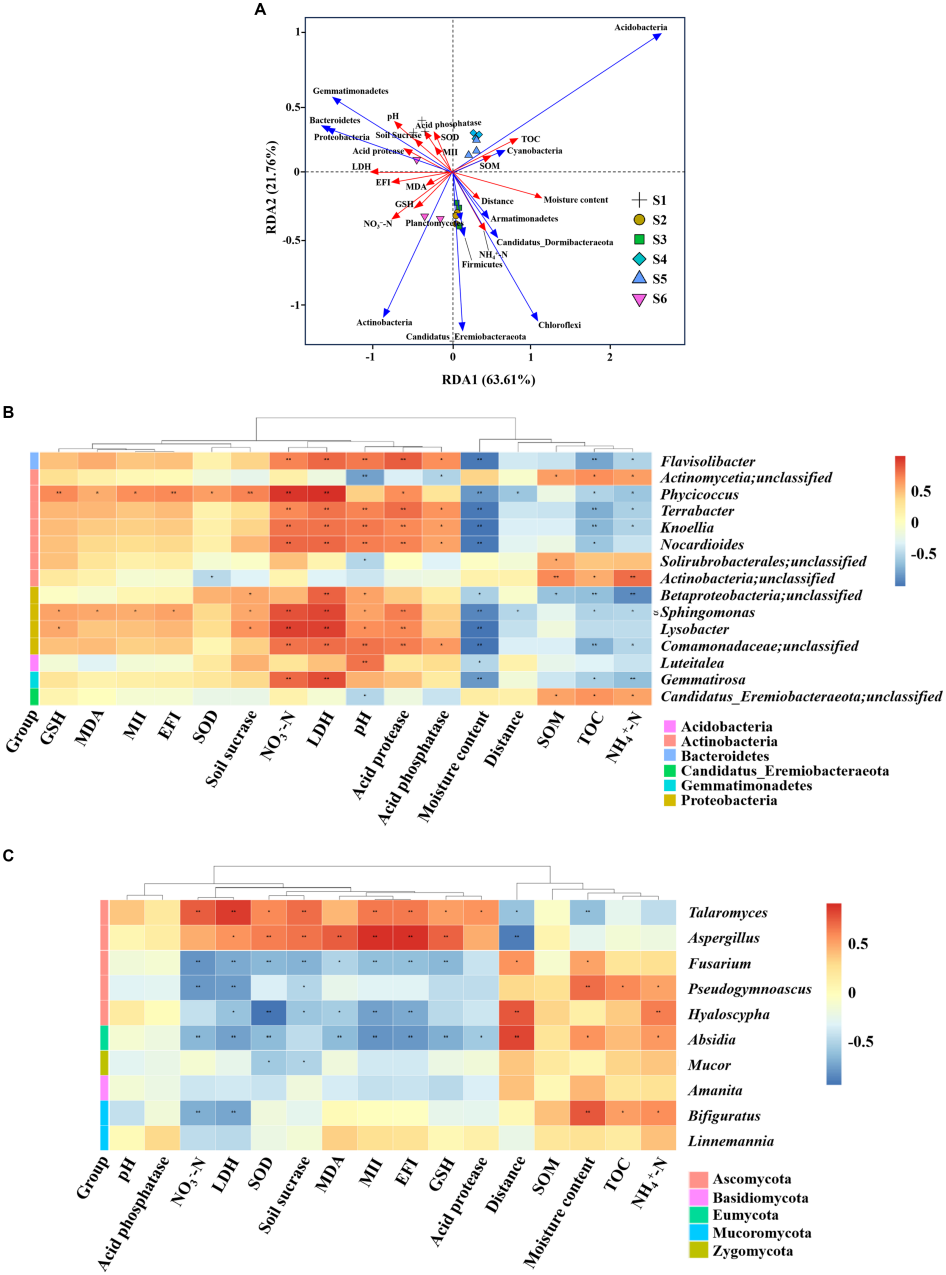
Correlation analysis between microorganisms and soil physicochemical properties across sites S1–S6. **(A)** Redundancy analysis of microbial communities with environmental factors. **(B,C)** Pearson correlation analyses between microbial communities and environmental factors. The distance between soil sample points signifies the similarities and differences in functional composition among samples. The projected distance from the sample point to the environmental factor indicated the extent to which the sample was influenced by the environmental factor. The closer the projection line, the more similar the impact of the environmental factor on the two samples. The angle between environmental factors/species denotes the positive and negative correlations between environmental factors/species. NH_4_^+^-N, ammonium nitrogen; MII, magnetic induction intensity; SOM, soil organic matter; TOC, total organic carbon; NO_3_^−^-N, nitrate nitrogen; EFI, electric field intensity; MDA, malondialdehyde; GSH, glutathione; SOD, superoxide dismutase; LDH, lactate dehydrogenase. Significance levels: *ρ < 0.05; **ρ < 0.01.

Environmental factors exerted a substantial influence on microbial communities and their functions. RDA served as a valuable tool for expressing the correlation between environmental factors and microbial communities. As illustrated in Figure 5A, the RDA1 and RDA2 axis accounted for 63.61 and 21.76%, respectively, of the total variation in microbial community composition and soil properties at the phylum level. The RDA model, based on soil microbial phylum-level data, effectively differentiated soils from various collection sites, corroborating our NMDS results. Specifically, the S1 site, situated nearest to the electric power substation, exhibited discernible associations with NO_3_^−^-N, SOM, EFI, MII, pH and soil enzymes, including SOD, GSH, soil sucrase, LDH, acid phosphatase, acid protease, MDA, but was negatively correlated with distance, moisture content and NH_4_^+^-N. However, the S3 and S4 sites were opposite. The S4 and S5 sites exhibited negatively correlated with EFI, NO_3_^−^-N, MDA and GSH. Cluster analysis revealed notable correlations between (Acidobacteria, Cyanobacteria) and TOC, SOM, moisture content, distance, MII, pH and SOD. Actinobacteria was positively correlations with NH_4_^+^-N, NO_3_^−^-N, EFI, GSH, MDA, LDH and distance. Proteobacteria and Bacteroidetes demonstrated positive correlations with pH, SOD, NO_3_^−^-N, MII, EFI and the aforementioned soil enzymes. Additionally, RDA of the correlation between environmental factors and microbial communities at the genus was provided in Supplementary Figure S3.

Pearson’s correlation analyses were further conducted to elucidate the effects of the 16 environmental factors on the microbial communities at the genus level (see the phylum level in Supplementary Figure S4), showcasing distinct abundances at the S1 site compared to other sites. It was observed that *Cyanobacteria* had a higher abundance at the S1 site, showing significant negative correlations with MII and EFI (*ρ* < 0.05) and significant positive correlations with distance (*ρ* < 0.05). Among the 15 bacteria genera (Figure 5B), four *Actinobacteria* genera, three *Proteobacteria* genera, and one *Gemmatimonadetes* genus demonstrated significant positive correlations with NO_3_^−^-N (*ρ* < 0.05), whereas one unidentified *Actinobacteria* genus, one unidentified *Candidaus_Eremiobacteraeota* genus, and one unidentified Actinobacteria genus exhibited significant positive correlations with NH_4_^+^-N (*ρ* < 0.05). Notably, bacteria that were significantly negatively correlated with moisture content included four *Actinobacteria* genera, four *Proteobacteria* genera, one unidentified *Gemmatimonadetes* genera, one *Acidobacteria* genus and one *Bacteroidetes* genus. Bacteria showing significant positive associations with pH included four *Proteobacteria* genera, one *Actinobacteria* genera, one unidentified *Gemmatimonadetes* genus, one *Bacteroidetes* genus. Additionally, Four Actinobacteria genera, one *Bacteroidetes* genus, three *Proteobacteria* genera, and one *Gemmatimonadetes* genus (*Gemmatirosa*) displayed a negative correlation with TOC (*ρ* < 0.05). Most importantly, *Actinobacteria* genus (*phycicoccus*) and *Proteobacteria* genera (*Sphingomonas*) exhibited a significant positive correlation (*ρ* < 0.05) with MII, EFI and distance. Regarding soil fungi (Figure 5C), those displaying a positive correlation with NH_4_^+^-N and moisture content included one *Ascomycota* genus, one *Mucoromycota* (*Bifiguratus*) and one *Eumycota* genus (*Absidia*), while those with a negative correlation with NO_3_^−^-N included two *Ascomycota* genus, one *Mucoromycota* (*Bifiguratus*) and one *Eumycota* genus (*Absidia*). Interestingly, *Absidia*, *Hyaloscypha*, and *Fusarium* demonstrated a significantly negative correlation with MII and EFI, while *Aspergillus* exhibited the opposite trend.

## 4 Discussion

### 4.1 Influence of soil collection site on soil properties

Soil, a vital component of an ecosystems, serves as the material foundation for plant and microbial survival. Its physicochemical attributes dictate the structural compositions of plant and microbial communities (Yang and Hu, 2021). As indicated by Wu et al. (2019), sites with lower moisture content and higher pH could experience upward movement of soluble salts from the deeper soil layers. Higher soil pH through reduced H^+^ release by roots and organic matter lead to lower soil TOC level (Hong et al., 2018), which was further accelerated by soil moisture content, which was known to enhance soil microbial activities influencing SOM mineralization and decomposition (Huang et al., 2013; Zhang et al., 2019; Cao et al., 2021). This was all exemplified by the conditions at the S1 site and other sites (Table 1). Notably, TN levels at the S1 site exceeded those at other sites (Table 1), possible attributed to the presence of strong MII and EFI, significant factors influencing microbial community assembly (Aksoy et al., 2010). Despite the absence of waste discharge typical of coal-fired power plants (Sun et al., 2024), the operation of electric power substation impacted soil physicochemical properties.

### 4.2 Influence of collection site on the composition of soil microbial communities

Across all soil samples, *Acidobacteria*, *Proteobacteria*, *Actinobacteria* and *Chloroflexi* emerged as dominant phyla (Figure 3A), aligning with findings from soils neighboring coal-fired power plants (Sun et al., 2024) as well as mining and smelting areas (Liu B. et al., 2021). However, NMDS and PCoA analysis indicated of distinct distribution patterns for soil bacterial community (Figure 2; Supplementary Figure S1). Particularly, the S1 site exhibited significantly higher abundances of *Proteobacteria* and *Acidobacteria* and lower abundances of *Chloroflexi* compared to other sites (Supplementary Table S1), which contradicted observations for coal-fired power plants (Sun et al., 2024). These characteristics indicated a higher capacity of reducing nitrogen loss and a diminished capability for autotrophic denitrification (Zhou et al., 2019; Ge et al., 2020; Tao et al., 2022; Wang et al., 2023; Wei et al., 2023). At the bacterial genus level, soil properties explained 85.5% of the variation in soil microorganism composition (Supplementary Figure S3), and discernible differences were evident among the 6 sites (Figure 3B). Bacterial taxa that were more enriched at the S1 site relative to other sites (Figure 3C), contained: (1) the genus *Unclassified_Comamonadaceae* (Sotres et al., 2016), *Lysobacte*r (Iwata et al., 2010), and *Sphingomonas* (Yang et al., 2014), known for their proficiency in facilitating superior nitrogen conversion. (2) Other microbial entities such as *Flavisolibacter* (Hernández-Guzmán et al., 2022), *Gemmatirosa* (Liu B. et al., 2021; Liu Z. et al., 2021; Sun et al., 2023), *Terrabacter* (Kruglova et al., 2017), and *Phycicoccus* (Yang et al., 2023), associated with nitrogen metabolism, along with the genera like *Nocardioides*, the functions of which were yet to be elucidated in the biogeochemical cycle. At the species level, despite an enrichment of *Sphingomonas mesophila* (Li et al., 2019) and *Sphingomonas edaphi* (Kim et al., 2020) was observed at the S1 site, their precise roles remained ambiguous. Nevertheless, most bacteria did not show statistically significant correlations with distance, as well as MII and EFI.

Reports regarding the composition and structure of soil fungal communities in the vicinity of electric power substation are notably limited. In this investigation, the fungal communities at the phylum level were predominantly constituted by *Basidiomycota* and *Ascomycota* in all soil samples (Figure 3D), a pattern consistent with their ubiquity in most soils (Shary et al., 2007). The prevalence of *Ascomycota* might be associated with its capacity to degrade cellulose and hemicellulose (Shary et al., 2007; Yang et al., 2023), while the phyla *Basidiomycota* and *Mucoromycota* had been reported to be linked to the degradation of complex lignocelluloses (Lundell et al., 2010; Huhe et al., 2017). Interestingly, in contrast to bacteria, several fungal genera such as *Talaromyces*, *Aspergillus*, *Fusarium*, *Hyaloscypha* and *Absidia* exhibited significant associations with MII, EFI and distance, suggesting that the influence of electric power substation operations on soil fungal communities surpasses that on bacterial populations.

### 4.3 Influence of soil collection site on the nitrogen processing

Nitrogen, a crucial limiting nutrient in ecosystems, plays a fundamental role in ecosystem productivity, where nitrogen fixation serves as a key mechanism for microorganisms to acquire nitrogen resources. The phyla *Proteobacteria* (42.64%) and *Gemmatimonadetes* (10.35%) from Supplementary Figure S4A demonstrated significant negative correlations with NH_4_^+^-N, displaying higher abundance at the S1 site characterized by lower soil moisture content (26.91%) and higher levels of MII and EFI. As revealed by RDA analysis, NH_4_^+^-N levels were negatively correlated with MII and EFI but positively correlated with distance. Given that nitrogen fixation predominantly occurs in anaerobic environments, the relative lower moisture content at the S1 site may impede soil nitrogen fixation (Zhang et al., 2020), which could explain the observed lower relative abundance of nitrogen fixation genes shown in Figure 4 and the reduction in NH_4_^+^-N content at this site. While nitrification, a key microbial nitrogen-loss pathway, displayed a positive correlation with MII and EFI, with genes associated with nitrification function showing increased abundance at the S1 site, likely contributing to the observed rise in NO_3_^−^-N levels post-operation of the electric power substation, although the mechanisms governing bacterial responses to electromagnetic fields of varying intensities remain unclear, impeding predictions of microbial behavior (Beretta et al., 2019).

## 5 Conclusion

This study employed metagenomic sequencing to investigate the relationships between microbial communities and environmental variables in soils located at varying distances from an electric power substation, which generated MII and EFI. Noteworthy discrepancies in soil physicochemical properties, as well as in the diversity, composition and structure of soil fungal communities, were evident under the influence of the electric power substation, while such distinctions were not observed in soil bacterial communities. Moisture content and pH influenced the soil bacterial communities, whereas the impact of anthropogenic disturbance linked to MII and EFI was pronounced in the immediate vicinity of the electric power substation, yet minimal as the sampling sites were distanced from the substation. Moreover, the genes associated with nitrogen fixation and nitrification functions were found to correlate with the observed levels of NH_4_^+^-N and NO_3_^−^-N. The soil fungal communities displayed greater sensitivity to the electric power substation operation compared to bacteria, and their dynamics had a direct and significant impact on microbial community diversity within the substation ecosystem.

## Data availability statement

The datasets presented in this study can be found in online repositories. The names of the repository/repositories and accession number(s) can be found in the article/Supplementary material.

## Author contributions

Z-XX: Data curation, Investigation, Methodology, Writing – original draft. BZ: Investigation, Visualization, Writing – original draft. SC: Data curation, Methodology, Writing – original draft. SX: Data curation, Formal analysis, Validation, Writing – original draft. L-GJ: Formal analysis, Validation, Writing – original draft. XL: Data curation, Validation, Visualization, Writing – review & editing. Y-FW: Validation, Visualization, Writing – original draft. L-XY: Conceptualization, Funding acquisition, Project administration, Supervision, Writing – review & editing.
