# Supplementary material for: Soil microbial community composition and nitrogen enrichment responses to the operation of electric power substation
Source: Front Microbiol. 2024 Aug 20;15:1453162. doi: 10.3389/fmicb.2024.1453162 (PMC11368844; doi:10.3389/fmicb.2024.1453162)
Supplement: Supplementary file 1 [file Data_Sheet_1.pdf]

Supplementary Material

1 Determination of NO_2^--N, NH_4^+-N and the total nitrogen

NO_2^--N measurement. 0.465 mol of p-aminobenzenesulfonamide was dissolved in 1 L of water solution that contained 200 mL of phosphoric acid solution (85%wt). Next, 20 mL of above solution was mixed homogenously with 20 mL of N-(1-Naphthyl)ethylenediamine dihydrochloride (0.015 M) and 20 mL of phosphoric acid (85%wt) to generate the color developing agent. 1 mL of soil supernatant was transferred into a 25 mL volumetric flask containing a 0.23 mL of color developing agent (see details in SI Section 1) and mixture homogenously. This was stand for over 1 h and measured at 543 nm using a UV-vis spectrophotometer (TU-1810, Bejing Purkinje General Instrument Co. Ltd., China) to quantitatively calculate its content according to its standard curve.

NH_4^+-N measurement. 10 mL of soil supernatant was transferred into a 50 mL volumetric flask containing a 20 mL of another color developing agent (15 mL of 0.74 M C_6H_5OH + 15 mL of 2.7 mM $Na_2[Fe(CN)_5NO]\cdot 2H_2O$ + 360 mL of H_2O). It was mixed homogenously and was stand for 15 minutes. 1 mL of 0.02 M $C_3Cl_2N_3NaO_3\cdot 2H_2O$ (dissolved in 0.952 M $C_6H_5Na_3O_7\cdot$2H2O + 0.55 M NaOH solution) was immediately added into the above mixture and was stand at 25°C for over 5 h. It was finally measured at 630 nm using a UV-vis spectrophotometer to quantitatively calculate its content according to its standard curve.

Total nitrogen measurement. 0.01 g of soil was air-dried, ground, filtered through a 100 mesh sieve, and subsequently transferred to a 10 mL of ammonia-free water. 5 mL of alkaline potassium persulfate solution was quickly added into the above solution. It was sealed tightly with cloth and rope, and was further transferred to a steam sterilizer at 121°C for 1 hour. After cooled to room temperature, 1.0 mL of HCl (1+9) was added into each mixture above and this was further diluted to a 25 mL volume with ultrapure water. Finally, this mixture was measured at 220 nm and 275 nm using a UV-vis spectrophotometer. Additionally, we replaced 0.01 g of soil sample with different concentrations of KNO_3 solution and 10 mL of ammonia-free water (blank test solution) to determine the amount of nitrogen in these solutions, respectively. Finally, we plotted a standard curve and another calibration curve based on corrected absorbance difference (*Ar*) and the corresponding nitrogen concentration to determine the content of the total nitrogen. *Ar* was calculated as followed:

$$A_{(blank\ test\ solution)}=A_{(blank\ test\ solution\ at\ 220\ nm)} - A_{(blank\ test\ solution\ at\ 275\ nm)}$$

$$A_{(calibration)}=A_{(calibration\ at\ 220\ nm)} - A_{(calibration\ at\ 275\ nm)}$$

$$Ar=A(_{calibration}) - A_{(blank\ test\ solution)}$$

2 Determination of the total carbon

Prior to experimentation, soil samples were finely ground to a particle size of 100 mesh (0.15 mm). The quantification of total carbon (TC) and total organic carbon (TOC) was carried out utilizing a total organic carbon analyzer (Multi N/C 3100, Analytikjena, Germany), employing $CaCO_3$ (C content = 120 mg/g) as standard reagent.

3 Determination of soil enzymes

Soil enzyme activity, encompassing malondialdehyde (MDA), superoxide dismutase (SOD), glutathione (GSH), lactate dehydrogenase (LDH), acid protease, acid phosphatase, and soil sucrase were assessed using enzyme-linked immunosorbent assay kits sourced from GenHunter, USA. A solid-phase antibody was generated by coating a microtiter plate with the corresponding purified enzyme antibody. The specific enzyme, in turn, was sequentially introduced into the microtiter wells coated with monoclonal antibody. Subsequently, the enzyme bound to the HRP-labelled enzyme antibody, forming an antibody-antigen-enzyme-labelled antibody complex. This complex underwent thorough washing, followed by exposure to the substrate TMB (3,3',5,5'-tetramethylbenzidine) to initiate color development. The TMB, acted upon by HRP enzyme, transitioned from blue to the final yellow color under acidic conditions. The color intensity exhibited a positive correlation with the enzyme concentration in the sample. Absorbance at 450 nm was measured using an enzyme marker, and the enzyme concentration in the sample was determined from a standard curve.

4 Function notes

The KEGG annotation was conducted using Diamond against the Kyoto Encyclopedia of Genes and Genomes database (http://www.genome.jp/keeg/, version 94.2) with an e-value cutoff of $1e^{-5}$ [1].

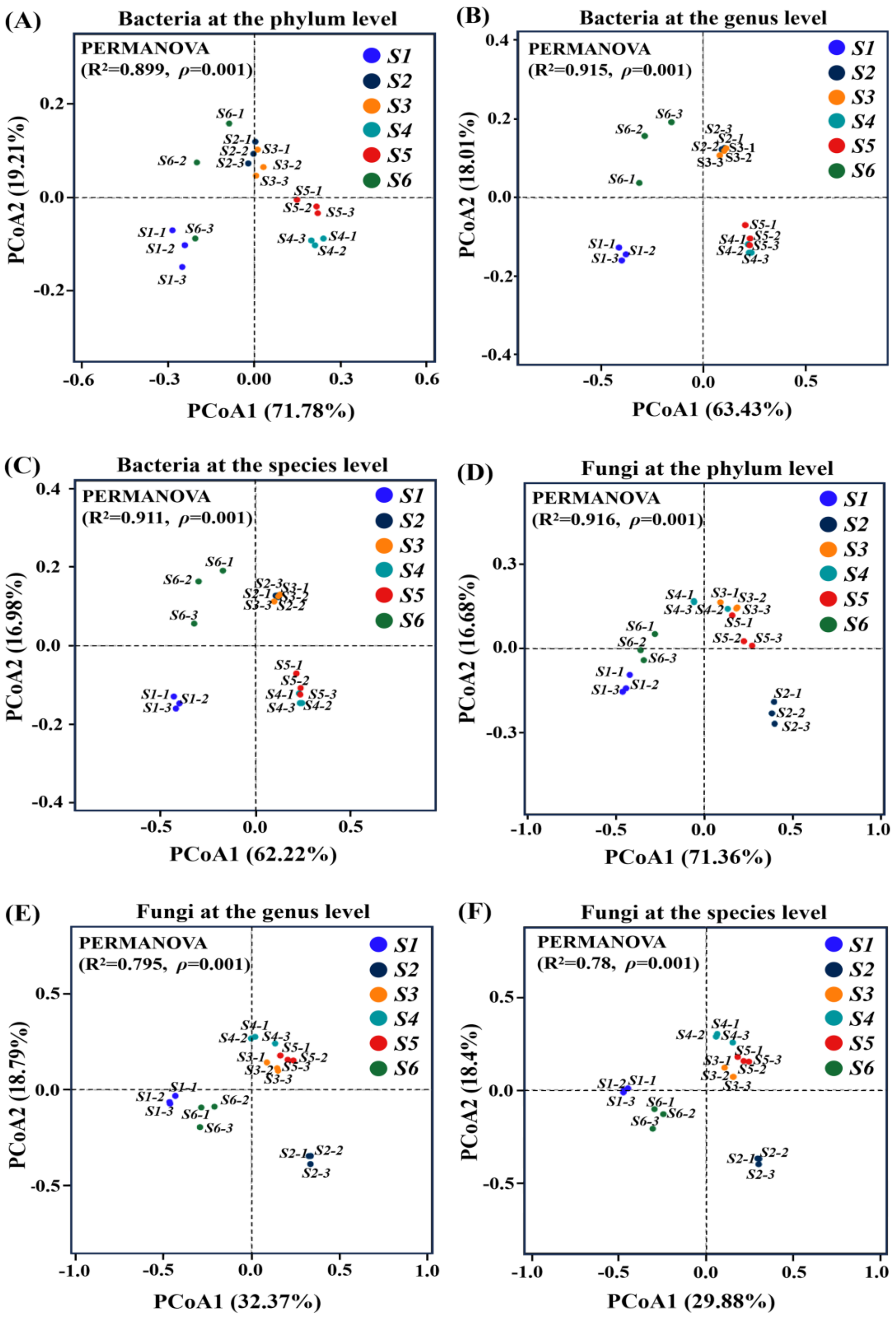

Supplementary Figure 1. PCoA plots of bacteria and fungi based on different soil samples from the S1–S6 sites and PERMANOVA tests of the differences in bacterial and fungi community structure based on Bray-Curtis distance.

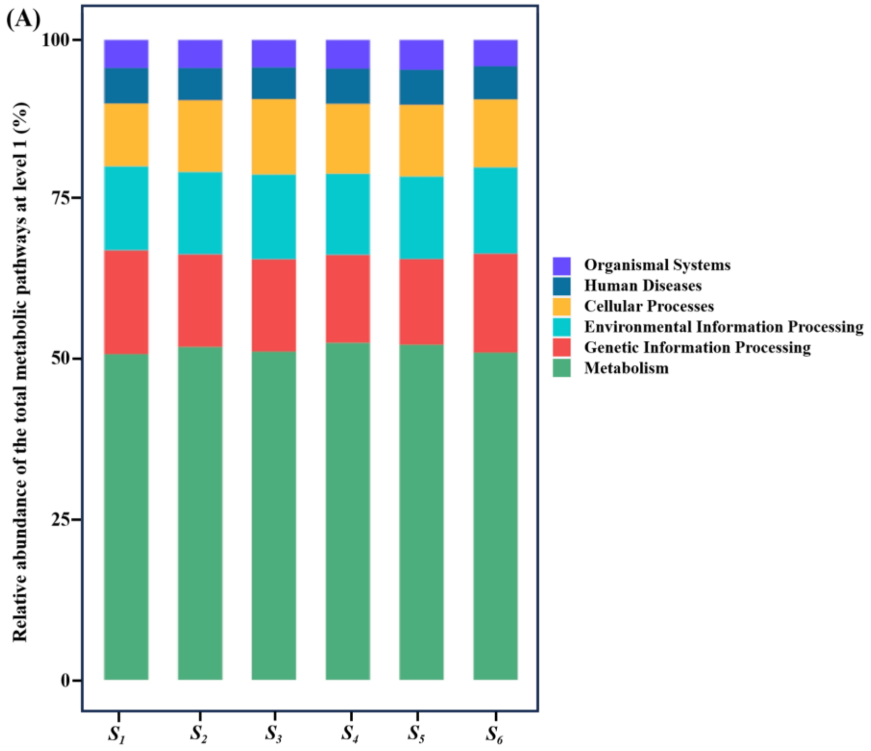
(A)
Relative abundance of the total metabolic pathways at level 1 (%)
100
75
50
25
0
S_1
S_2
S_3
S_4
S_5
S_6
Organismal Systems
Human Diseases
Cellular Processes
Environmental Information Processing
Genetic Information Processing
Metabolism

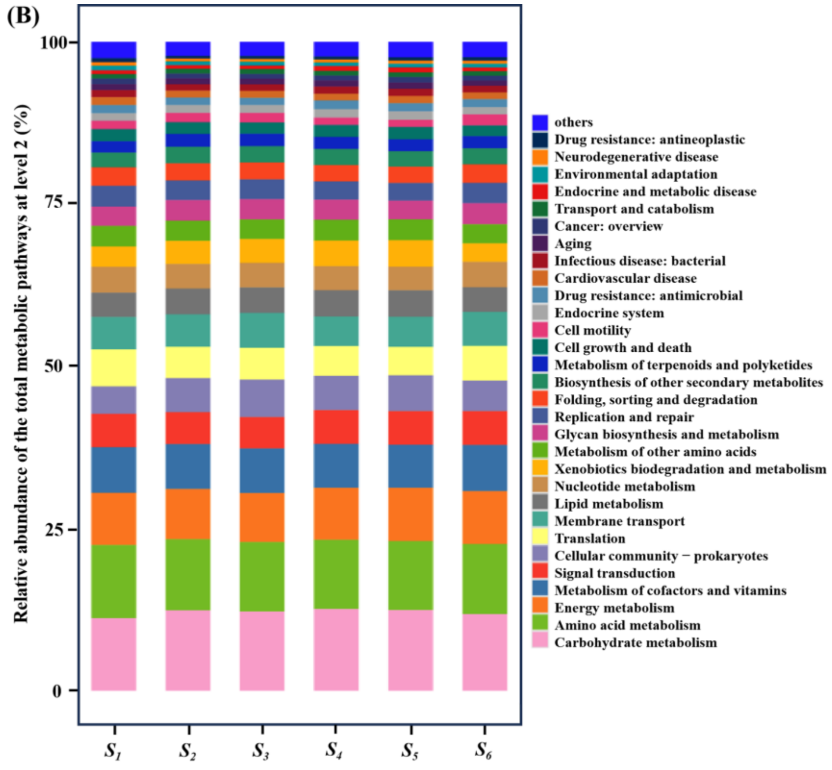
(B)
Relative abundance of the total metabolic pathways at level 2 (%)
100
75
50
25
0
S_1
S_2
S_3
S_4
S_5
S_6
others
Drug resistance: antineoplastic
Neurodegenerative disease
Environmental adaptation
Endocrine and metabolic disease
Transport and catabolism
Cancer: overview
Aging
Infectious disease: bacterial
Cardiovascular disease
Drug resistance: antimicrobial
Endocrine system
Cell motility
Cell growth and death
Metabolism of terpenoids and polyketides
Biosynthesis of other secondary metabolites
Folding, sorting and degradation
Replication and repair
Glycan biosynthesis and metabolism
Metabolism of other amino acids
Xenobiotics biodegradation and metabolism
Nucleotide metabolism
Lipid metabolism
Membrane transport
Translation
Cellular community − prokaryotes
Signal transduction
Metabolism of cofactors and vitamins
Energy metabolism
Amino acid metabolism
Carbohydrate metabolism

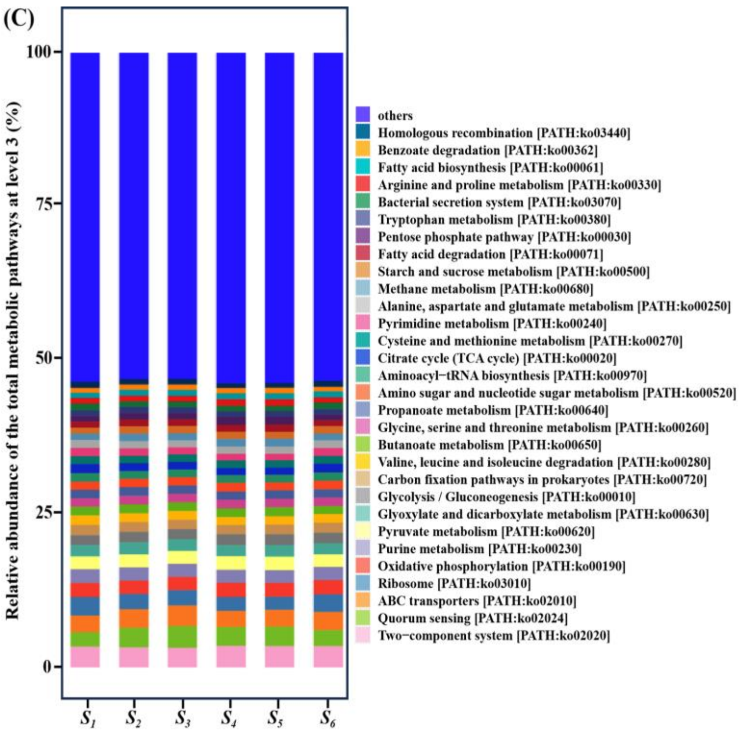

Supplementary Figure 2. The relative abundance of the total metabolic pathways at (A) level 1, (B) level 2 and (C) level 3 of S1-S6.

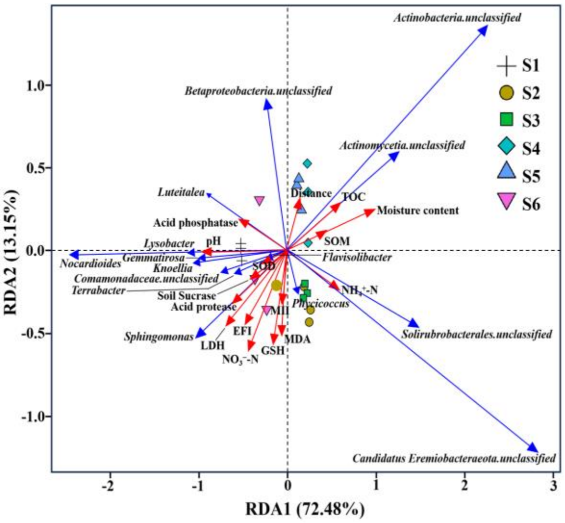

Supplementary Figure 3. Correlation analysis between microorganisms and soil physicochemical properties from the S1–S6 sites through Redundancy analysis. NH_4^+-N, ammonium nitrogen; MII: magnetic induction intensity; SOM, soil organic matter; TOC, total organic carbon; NO_3^--N, nitrate nitrogen; EFI, electric field intensity; MDA, malondialdehyde; GSH, glutathione; SOD, superoxide dismutase; LDH, lactate dehydrogenase.

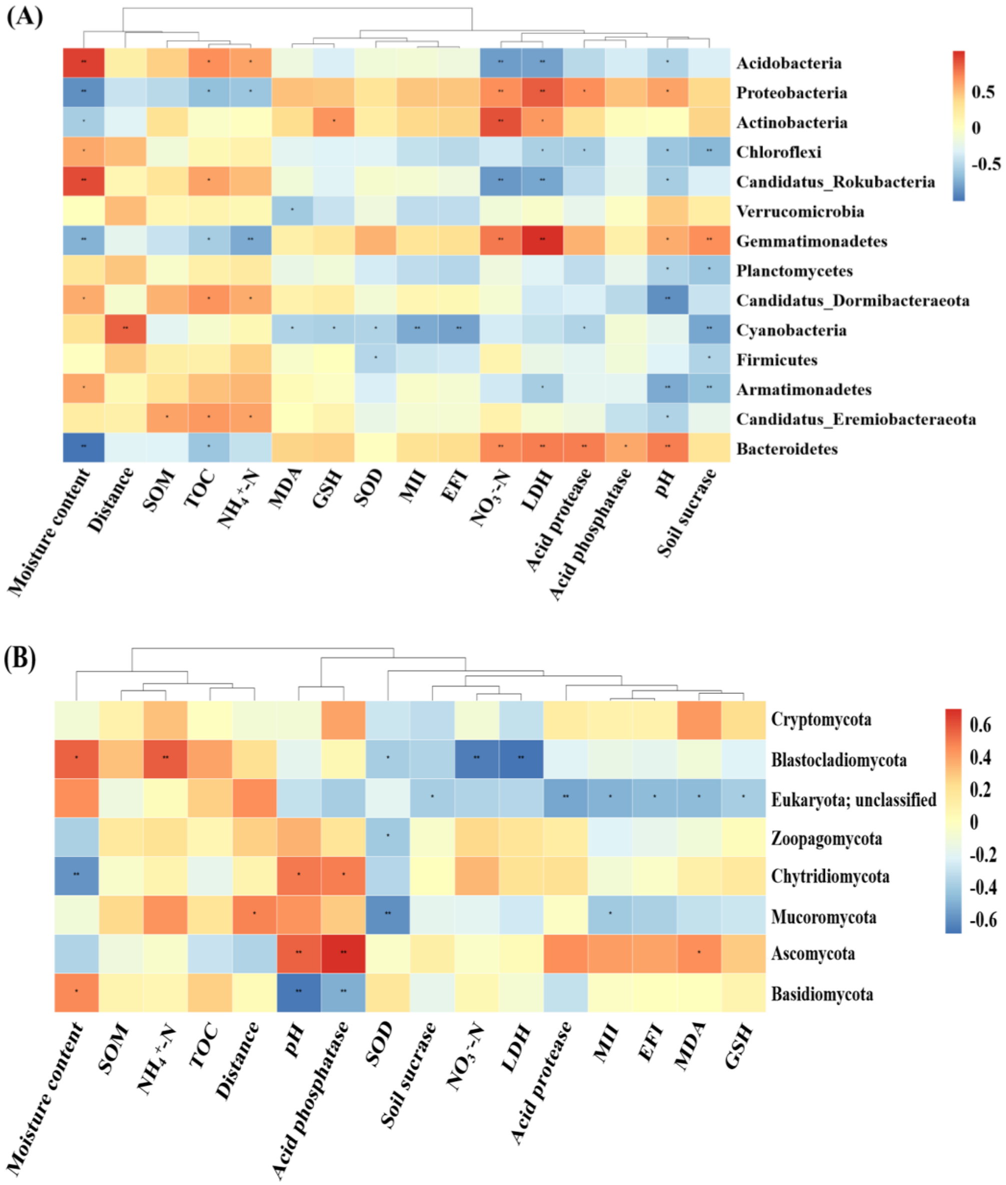

Supplementary Figure 4. (A) Pearson correlation analyses between bacteria communities and environmental factors at the phylum level. (B) Pearson correlation analyses between fungal communities and environmental factors at the phylum level. The distance between soil sample points signifies the similarities and differences in functional composition among samples. The projected distance from the sample point to the environmental factor indicated the extent to which the sample was influenced by the environmental factor. The closer the projection line, the more similar the impact of the environmental factor on the two samples. The angle between environmental factors/species denotes the positive and negative correlations between environmental factors/species. NH_4^+-N, ammonium nitrogen; MII: magnetic induction intensity; SOM, soil organic matter; TOC, total organic carbon; NO_3^--N, nitrate nitrogen; EFI, electric field intensity; MDA, malondialdehyde; GSH, glutathione; SOD, superoxide dismutase; LDH, lactate dehydrogenase. Significance levels: $*\rho < 0.05$; $**\rho < 0.01$.

Supplementary Table 1. Plot details

Plot	GPS information	Altitude (m)	Distance from the substation (m)	Relative abundance on phylum level	Relative abundance on genus level
**S1**	26°20′52.9848N 119°5′8.356632″E	778.45	5	Proteobacteria (42.64%), Actinobacteria (21.80%), Acidobacteria (13.03%), Gemmatimonadetes (10.35%), Bacteroidetes (6.21%), Verrucomicrobia (1.70%), Chloroflexi (1.55%)	*Nocardioides, Luteitalea, Gemmatirosa, Flavisolibacter, Lysobacter, Comamonadaceae;unclassified, Sphingomonas, Knoellia, Terrabacter, Solirubrobacter, Phycicoccus*
**S2**	26°20′51.5657N 119°5′12.7017″E	788.33	20	Actinobacteria (29.26%), Proteobacteria (23.44%), Acidobacteria (23.30%), Chloroflexi (8.77%), Candidatus_Eremiobacteraeota (4.14%), Candidatus_Dormibacteraeota (1.74%)	*Candidatus_Eremiobacteraeota; unclassified, Blastococcus, Actinobacteria;unclassified, Sinomonas, Solirubrobacterales;unclassified, Actinomycetia;unclassified, Streptomyces* *Pseudonocardiales;unclassified*
**S3**	26°20′48.8314N 119°5′13.879824″E	790.79	30	Acidobacteria (25.10%), Proteobacteria (24.68%), Actinobacteria (23.19%), Chloroflexi (10.94%), Candidatus_Eremiobacteraeota (2.30%), Bacteroidetes (1.69%)	*Armatimonadetes;unclassified, Candidatus_Dormibacteraeota; unclassified, Granulicella, Arthrobacter, Blastococcus, Actinobacteria;unclassified*
**S4**	26°9′16.6605N 119°4′58.0894″E	770.66	50	Acidobacteria (51.32%), Proteobacteria (17.10%), Actinobacteria (14.91%), Chloroflexi(5.89%), Verrucomicrobia (2.57%), Candidatus_Rokubacteria (1.75%)	*Gammaproteobacteria;unclassified, Acidobacteria;unclassified, Actinomadura, Edaphobacter, Actinomadura*
**S5**	26°20′42.2378N 119°5′11.58144″E	775.48	100	Acidobacteria (44.29%), Proteobacteria (20.29%), Actinobacteria (13.48%), Chloroflexi (11.66%), Candidatus_Rokubacteria (1.62%), Verrucomicrobia (1.48%)	*Acidobacteria; unclassified, Ktedonobacter, Ktedonosporobacter, Mycobacterium, Thermogemmatispora*
**S6**	26°20′37.8594N 119°5′6.0000″E	773.27	230	Proteobacteria (29.26%), Actinobacteria (25.45%), Acidobacteria (11.31%), Chloroflexi (9.86%), Gemmatimonadetes (6.34%), Bacteroidetes(5.46%)	*Catenulispora, Cronobacter, Rhodanobacter, Chloroflexi;unclassified, Micromonospora, Gemmatirosa, Flavisolibacter*

Supplementary Table 2. Sequencing data

Sample	Total Reads	Clean Reads	Percentage	Clean bases	GC content	%>Q20	%>Q30
S1-1	70,698,204	69,848,674	98.80%	10,471,818,994	65.47%	97.71%	93.95%
S1-2	69,541,096	68,696,650	98.79%	10,296,793,185	65.07%	97.71%	93.95%
S1-3	72,884,892	72,080,698	98.90%	10,806,596,767	65.35%	97.55%	93.17%
S2-1	74,681,982	73,922,462	98.98%	11,083,678,204	63.79%	97.47%	92.92%
S2-2	70,878,216	70,174,308	99.01%	10,518,281,214	63.40%	97.81%	93.78%
S2-3	76,859,302	76,153,354	99.08%	11,415,966,121	63.39%	97.74%	93 55%
S3-1	70,093,350	69,212,972	98.74%	10,370,970,833	63.22%	97.53%	93 52%
S3-2	70,599,706	69,875,244	98.97%	10,473,755,792	63.00%	97.78%	93 65%
S3-3	75,791,892	75,055,938	99.03%	11,251,533,684	63.03%	97.67%	93.37%
S4-1	69,070,716	68,404,936	99.04%	10,253,097,506	60.73%	97.79%	94.03%
S4-2	67,715,992	67,031,004	98.99%	10,049,994,769	61.25%	97.55%	93.10%
S4-3	78,630,186	77,815,096	98.96%	11,662,801,187	61.39%	97.68%	93.43%
S5-1	69,513,354	68,835,032	99.02%	10,318,781,476	61.63%	97.91%	94.31%
S5-2	68,834,862	68,165,852	99.03%	10,219,446,186	61.25%	97.58%	93.14%
S5-3	74,780,466	74,104,920	99.10%	11,107,549,382	61.25%	97.73%	93.49%
S6-1	70,335,916	69,467,042	98.76%	10,412,673,210	63.94%	97.64%	93.78%
S6-2	75,770,016	75,217,172	99.27%	11,278,602,111	64.13%	98.07%	94.45%
S6-3	68,931,068	67,721,540	98.25%	10,148,984,946	65.26%	97.85%	93.98%

Reference

1. Buchfink, B., Xie, C., and Huson, D.H. (2014). Fast and sensitive protein alignment using DIAMOND. Nat. Methods. 12, 59–60. https://doi.org/10.1038/nmeth.3176
